# Responsibility for scientific misconduct in collaborative papers

**DOI:** 10.1007/s11019-017-9817-7

**Published:** 2017-12-08

**Authors:** Gert Helgesson, Stefan Eriksson

**Affiliations:** 1 Stockholm Centre for Healthcare Ethics (CHE), Department of Learning, Informatics, Management and Ethics, Karolinska Institutet, Stockholm, Sweden; 2 Centre for Research Ethics and Bioethics (CRB), Department of Public Health and Caring Sciences, Uppsala University, Uppsala, Sweden

**Keywords:** Accountability, Authorship, Research ethics, Research integrity, Responsibility, Scientific misconduct

## Abstract

This paper concerns the responsibility of co-authors in cases of scientific misconduct. Arguments in research integrity guidelines and in the bioethics literature concerning authorship responsibilities are discussed. It is argued that it is unreasonable to claim that for every case where a research paper is found to be fraudulent, each author is morally responsible for all aspects of that paper, or that one particular author has such a responsibility. It is further argued that it is more constructive to specify what task responsibilities come with different roles in a project and describe what kinds of situations or events call for some kind of action, and what the appropriate actions might be.

## Introduction

Research collaboration has become increasingly important in the last decades, not least because of the complexity of much of modern research and the need to involve different competencies in order to successfully bring projects to completion. Studies show that the average number of authors per paper in medicine and the natural sciences has increased considerably over time. For instance, in 2001, Rennie reported that there had been a three-fold increase in the average number of authors on papers in *The Lancet* since the 1950s (Rennie 2001). In 2007, Greene reported that four times as many authors populated articles and letters to the editor in *Nature* compared to 1950 (Greene 2007). Since then, papers have continued to report an increasing number of authors per paper in a variety of journals (Modi et al. 2008; Rahman and Muirhead-Allwood 2010; McSorley 2011; Ojerholm and Swisher-McClure 2015; Schrock et al. 2016; Offord 2017).

With a growing number of co-authors, it arguably becomes increasingly difficult to identify those who should be held morally responsible in cases of scientific misconduct. While such identification may be fairly simple and straight forward in small projects within one field, it becomes increasingly challenging as the number of collaborators, research sites, and areas of expertise increase.

The difficulties are not only related to the challenge of evaluating the contributions to the project of the individual researchers, but also to the fact that different researchers take on different roles in collaborations. For instance, in larger collaborations a centre and a periphery can usually be identified. Even when contributions are substantial enough to qualify for authorship according to the ICMJE authorship guidelines (ICMJE 2016), some researchers may have contributed little to the paper in whole. Even if properly informed about the main thrust of the paper and willing to take on a critical revision of the manuscript, they may regard themselves responsible only for their own specific contribution, particularly if they have not participated in planning the study and if other parts of the paper contain work in areas that lie beyond their own competence. However, some research ethical guidelines suggest, to the contrary, that all authors on a paper should be held morally responsible if the paper turns out to be fraudulent (see, e.g., ALLEA 2017).

Eggert has argued that “[a]uthorship conveys responsibility in that it implies the endorsement of the quality and integrity of the work performed” (Eggert 2011, p. 1). The question is how. Should all authors be held responsible for the misconduct, or only those who fulfil certain criteria related to their involvement at different stages of the paper? In this paper, we will explore the arguments relating to the moral responsibility of co-authors in case their paper turns out to involve misconduct.

We will restrict the discussion to responsibility in relation to scientific misconduct. Hence, we will not discuss authorship responsibilities in general, which also include, for instance, responsibility for research participants and proper data protection. We do not exclude that there may be situations where other people than those listed as authors (such as other researchers, superiors, technicians, statisticians, or administrative personnel) share in the responsibility for the scientific misconduct, but we will not discuss such cases here. Nor will the paper concern responsibilities of organizations, such as universities or companies where the researchers are employed.

The paper is structured as follows: First we explain our use of terminology. Thereafter we present what is said on authorship responsibilities in some research integrity guidelines. We thereafter discuss what is a reasonable view regarding ‘being fully responsible’ for the research and how it is presented in the paper, partly in relation to the different roles researchers may have in a project. We will also discuss holding everyone responsible for everything about the paper as a means to deter future misconduct. Finally, we discuss how research roles and associated responsibilities can be specified in order to promote a responsible attitude towards the scientific integrity of the paper.

## What we mean by ‘responsibility’

Different terms are used in discussions of responsibility, and sometimes different meanings are attached to the same words. ‘Responsibility’ can relate to tasks that have been assigned to the individual, as in “It is your responsibility to analyse those blood samples” (task responsibility). ‘Responsibility’ can also be used to identify someone’s causal role in an event (causal responsibility). Furthermore, ‘responsibility’ can be used to mean moral (or legal) responsibility or blameworthiness, as in “It is entirely your fault, so you are the one to blame—you are responsible, not anyone else” (Dworkin 1981; Goodin 1987).

A term similar in meaning to the latter is ‘culpability’, which is used for moral or legal responsibility, primarily to denote “direct involvement in the wrongdoing, such as through participation or instruction” (Arnone 2014). Since culpability is a legal term and therefore is likely to be interpreted in the light of the complexities and particularities of specific legal systems, we will not use it here.

Another term often used in discussions of moral responsibility is ‘accountability’. We will not try to pinpoint its exact relation to moral responsibility here, but accountability relates to an awareness and assumption of the role of being the one to blame if things go wrong. Still, ‘accountability’ often seems to be used interchangeably with ‘responsibility’. If a distinction can be maintained between (moral) responsibility and accountability, it seems to be the one between being the one whose fault it actually is and being the one who is or should be held responsible. The latter can be decided by convention, while settling the former requires normative analysis. Henceforth, we will maintain this distinction by using the expressions ‘being responsible’ and ‘being held responsible’. In line with what has just been said, one might argue that someone who is not seen as morally responsible for a certain outcome nevertheless should be held responsible—for some other reason than blameworthiness, such as having strict liability according to contract or law.

## The messages of central research integrity guidelines

Research integrity guidelines have quite a bit to say about what is expected of authors in terms of contributions and research performance, but much less about the responsibilities of co-authors in cases of scientific misconduct.

According to the Council of Science Editors (CSE), an international organization for science editors, “The ultimate reason for identification of authors and other contributors is to establish accountability for the reported work”. CSE specifies: “Authors are individuals identified by the research group to have made substantial contributions to the reported work and agree to be accountable for these contributions.” Each author is “accountable for the parts of the work he or she has done”. In the CSE *White paper on promoting integrity in scientific journal publications* (CSE 2012), wherefrom the quotes are taken, there are no suggestions that each author, or some author, is responsible for what other contributors do, although authors are expected to be aware of the collaborators’ contributions.

The *Recommendations for the conduct, reporting, editing, and publication of scholarly work in medical journals*, issued by the International Committee of Medical Journal Editors (henceforth the ICMJE guidelines), and the most influential authorship guidelines to this day, have through their different versions strongly stressed the idea that all authors of a paper should share not only scientific credit for the work but also responsibility for that paper (ICMJE 2016). This also explains why it has been insisted that all authors not only do some substantial contribution to the research, but also participate in the writing or critical revising of the paper, in order to qualify as authors—unless you read the paper critically, you cannot take responsibility for it (cf. Strange (2008, C567): “You can only assume responsibility if you were intellectually engaged in the work and in writing the manuscript”). But the exact message has varied between versions, from the view that each author is responsible for the entire paper to the view that each author is responsible at least for their own contribution. In the latest version, it is stated:

In addition to being accountable for the parts of the work that he or she has done, an author should be able to identify which co-authors are responsible for specific other parts of the work. In addition, authors should have confidence in the integrity of the contributions of their co-authors.

The fourth criterion for authorship further requires:

Agreement to be accountable for all aspects of the work in ensuring that questions related to the accuracy or integrity of any part of the work are appropriately investigated and resolved.

This implies that authors should not only know and take responsibility for their own contributions to the paper, but they should know enough about the collaboration as a whole to be able to say, for instance, who else were included in the collaboration and what they contributed—which implies being able to help identify who did what if, for instance, certain analyses turn out to be fraudulent.

It has been suggested to us on several occasions in conversation that the fourth criterion should be taken to imply that all authors have personal responsibility for all parts of the work, a view echoed in the literature (e.g. Kornhaber et al. 2015; Leventhal 2016). But this seems to be a reasonable interpretation only if one stops reading after the first occurrence of “the work”. The fourth criterion rather seems to say that each co-author must collaborate with misconduct investigators (for instance, by providing research protocols or relevant email conversations upon request) if their paper is called into question.

In a position statement developed at the 2nd World Conference on Research Integrity 2010,^1^ supported by the Committee on Publication Ethics (COPE) and published at the COPE website (Wager and Kleinert 2011), it is stated that research should be carried out in an ethical manner, should be sound and carefully executed, using appropriate methods, and should be presented in an honest, correct, and non-misleading way—but also that authors “should take collective responsibility for their work and for the content of their publications” (Section 1.4). Later in the position statement (Section 7.1) this proposal is specified as follows:

In most cases, authors will be expected to take joint responsibility for the integrity of the research and its reporting. However, if authors take responsibility only for certain aspects of the research and its reporting, this should be specified in the publication.

ALLEA (“All European Academies”, i.e., The European Federation of Academies of Sciences and Humanities), in its 2017 edition of *The European Code of Conduct and Research Integrity*, concurs with this understanding of authorship responsibility: “All authors are fully responsible for the content of a publication, unless otherwise specified.” The shared idea here seems to be that the primary option should be that all authors share the responsibility equally for the paper in case of scientific misconduct. The only circumstances under which not everyone should be held equally responsible are when limitations in this regard are specifically stated in the publication.

On the account that such specifications are rarely made (journals requiring a contribution statement are in a clear minority), there is disagreement between on the one hand the CSE and the ICMJE guidelines and on the other the COPE-supported position statement/ALLEA regarding the responsibility of the individual author. Where COPE/ALLEA requires everyone to assume equal responsibility for misconduct also when only a limited few are actually guilty, the CSE/ICMJE guidelines suggest that those who did it should assume responsibility for it. COPE/ALLEA can perhaps be read as promoting a practice where limitations in responsibility are standardly described in the paper by concerned authors, rather than favouring blaming all authors if the paper is fraudulent. This would be in line with the declaration, for instance, of the journal PLOS One (at http://journals.plos.org/plosone/s/authorship): “We expect that all authors will take public responsibility for the content of the manuscript submitted to PLOS. The contributions of all authors must be described.” But if this is what COPE/ALLEA mean, then they should say so.

Importantly, it is not clarified in any of these sources whether or not declarations of contributorship, as presently used by some journals, would be sufficient ground for differentiation in accountability. Rennie et al. (1997) argued that this would be the very point of contributorship statements and that they should be made compulsory. Let us conclude that a more widespread application of the practice of making contributorship declarations would support a practice of distinguishing between the different responsibilities of different authors.

## Everyone morally responsible for fraudulent papers?

So who is morally responsible for the fraud of a fraudulent paper? Potential answers are: “those who have done it”, “every author on the paper”, or “it depends…” with an added specification of what responsibility hinges on. None of the guidelines we have discussed suggest that all authors should unconditionally be held responsible for the entire paper. At most, everyone should be held responsible for everything about the paper unless it is specified beforehand who is responsible for what. But is this condition reasonable in cases where author contributions have not been specified beforehand? Why should everyone be held responsible if not everyone is responsible?

It is not a defensible position to hold every co-author responsible regardless of what has happened, even if the division of responsibility is not stated beforehand. Co-authors of scientific papers should not be held responsible for misconduct unless they

1. were personally involved in the actions constituting scientific misconduct, or
2. encouraged misconduct, or
3. knew about or suspected it without taking appropriate action.

The reason for this is that responsibility for research misconduct must be on par with the responsibility we assume in other cases of wrongdoing, and thus intention or recklessness needs to be present.

One consequence of this is that you should not be held responsible for your collaborators’ fraudulent behaviour if you were unaware of it and had no indications of what was going on. This can easily be the case in large collaborations where different groups make their contributions independent of one another, while a small group of researchers lead and orchestrate the work. This is not to say that researchers remain without any responsibility as long as they do not commit misconduct themselves, do not encourage others to do it, and remain ignorant of what their collaborators are doing. Every author shares the responsibility to be attentive to signs of misconduct and is under obligation to take some kind of action if they suspect fraudulent or too sloppy behaviour by collaborators in the study in which they participate. Every author also assumes a responsibility when publishing a paper to help rectify situations where their paper’s accuracy is questioned (ICJME 2016). However, on the whole it is not reasonable, nor ethically required, that collaborators spend time and effort scrutinizing what everyone else is doing to make sure it is scientifically and ethically sound, without any indications that this is needed.

## Someone responsible for everything about the paper?

If not everyone should be held fully responsible for the entire paper, i.e., responsible for all the research related to the paper and its presentation, then who should be? Obviously, responsibility may vary with the different roles collaborators have in a joint project. Researchers with a central role in the collaboration could indeed be responsible for having a certain overview of the project, which might include learning a bit about how new collaborators work, checking up on progress, discussing and dealing with encountered difficulties—all while keeping the question in mind whether what is going on is ethically sound. But is anyone fully responsible if no one has taken an active part in all the work included in the paper, i.e., in planning and study design, all data collection, and all different analyses?

Several authors (Rennie et al. 1997; Eggert 2011), guidelines (e.g., the ICMJE guidelines), and journals (e.g., *BMJ*) have proposed that there should be at least one researcher, the guarantor, who assumes full responsibility for the paper. Smith and Williams-Jones (2012) describe the guarantor as the researcher who “has overall responsibility (i.e., for the quality of the research findings and integrity of the research methods)”. The American Psychological Association states (at http://www.apa.org/research/responsible/publication/index.aspx): “The primary author assumes responsibility for the publication, making sure that the data are accurate, that all deserving authors have been credited, that all authors have given their approval to the final draft; and handles responses to inquiries after the manuscript is published.” *BMJ* states in its instructions to authors: “The guarantor accepts full responsibility for the work and/or the conduct of the study, had access to the data, and controlled the decision to publish.” The idea, in short, is to identify someone, or some, with responsibility for “the integrity of the work as a whole” (Eggert 2011, p. 197).

While this requirement may be justified for papers building on research carried out in small groups, where a senior researcher can be expected to have full control, or get it if needed, it is not so in large collaborations. First, a main reason for collaborating is the need for expertise only found outside one’s own group. In such cases, the PI for the project will likely not have the competence to monitor and verify the work of all collaborators. Secondly, even if they were capable of checking up on everything in this manner, it is questionable whether we want to see such practices in collaborative research, where the guarantors go through all the details of what the others have done in order to make sure things are as they should. We need to trust that they are, unless we have some indications to the contrary. To do otherwise would increase the cost of collaboration tremendously and in practice make most collaboration unattractive, with the likely consequence of slowing down scientific progress considerably. So while one or several researchers must be responsible (have the task responsibility) for the study as a whole—i.e., for the planning, how different parts of the collaboration fit in, who is expected to do what, etc.—no one must assume full moral responsibility for what collaborators do in the project.

## The deterring effect of holding all authors responsible

The discussion so far has built on the assumption that holding authors responsible should be tied to their actually being guilty of some kind of misconduct, either by acting fraudulently or by failing to act when realizing that someone else is misbehaving or is behaving suspiciously, or producing suspicious data. But another approach would be to use responsibility in a purely consequentialist way; that is, to hold researchers responsible to the extent that doing so has the best consequences. For instance, one could hold all authors fully responsible, also the innocent ones, because of the deterring effect and the overall better total outcome for the development of science of handling authorship responsibility this way. An example from *The Scientist* shows that many co-authors might be implicated if they collaborate with quite few fraudulent researchers: together the top eight researchers on the list of most individual retractions of published papers have more than 320 other researchers listed as authors on their problematic papers (Offord 2017).

Holding everyone responsible would be obviously unfair to the collaborating researchers who are not guilty of misconduct. However, fairness is of little concern to such an approach (at least in the short-term perspective)—instead focus lies on progress in research. While a steady increase in scientific collaboration has promoted development, it could be argued that it has also brought with it an increasing sloppiness regarding ethical standards, by making responsibility more diffuse. The sense that the researcher must take full responsibility has faded, not least because it is no longer practically possible. But that might have led to a deterioration in the attitude towards authorship responsibility altogether. By holding all authors responsible in case of scientific misconduct, the story goes, researchers are pressured to become much more careful about whom they collaborate with—and to set up control mechanisms to reduce the risks of being drawn into bad research practices (Offord 2017). This might contribute to better considered collaborations and more responsible authorship practices, which in turn might be beneficial to science in the longer run by increasing the quality and trustworthiness of published research. If it also reduces the proportion of undeserved publications, it might even have a positive long-term effect on fairness.

However, the deterrence argument as described here rests on the assumption that overall consequences in fact will be better. If this is not the case, the argument fails. Indeed, there are obvious actual and potential disadvantages with this kind of deterrence. Firstly, if successful, the ends are obtained by causing new wrongs, since innocent researchers will be treated as if guilty of misconduct. From a deontological perspective, this might be unacceptable even if the overall consequences are valuable. Secondly, there is the risk that scientists are provoked into bad behaviour if they perceive practices as unfair—as has been seen in cases of questionable judgements by funding agencies and ethical review committees (IRBs) (Keith-Spiegel et al. 2006; Martinson et al. 2006). Thirdly, there is an obvious risk that such a deterrence strategy will have a strongly negative effect on research collaboration—if punishments for scientific misconduct are severe enough, the cost of picking the wrong collaborator may be so serious that it is better to play it safe and not even try. To get a great deterrence effect, punishment needs to be harsh, but then the benefit–risk ratio for collaborating with researchers will make new collaborations less tempting.

It is difficult to tell what the consequences would be of using a deterrence policy as described above. As indicated, the consequences will likely depend to some extent on the severity of the punishment. However, it cannot be excluded that the negative effects of deterrence will be greater than the positive effects—i.e., that the loss of never initiated collaborations will be greater than the gain of reducing scientific misconduct. It seems to us that the burden of proof should lie with the defenders of this deterrence approach, since it involves unfair treatment of researchers.

## A modified deterrence approach

In the most extreme version of the deterrence approach, every co-author on fraudulent papers will be held responsible in every case—that is, also in cases where it has been shown who were involved in the misconduct and who were not. This is the most unfair version and also the version that is likely to deter the greatest number of researchers from collaborating.

A modified version would be one where everyone is held responsible in the absence of proof of who is indeed guilty of the misconduct. There are attractive aspects of such an approach. First, fraudulent researchers would not get away simply by blaming each other in the absence of clear evidence. Secondly, innocent collaborators who can show that they were not involved in the misconduct will not be held responsible. Thirdly, this approach would give co-authors incentives to document their contributions and perhaps also conversations and email discussions on important issues relating to the performance of the research. It would not be as deterrent and it would not be as unfair as the most extreme version. However, it would still be unfair in situations where some co-authors are not involved in misconduct, but cannot convincingly show that they are not, and are therefore held responsible. It would also go against the legal doctrine of presumption of innocence until proven guilty. Furthermore, its deterring effects are unclear. Still, one could argue that accepting to publish research papers lacking robust data management plans with clear traceability of responsibility by itself is worthy of criticism, not least because it gets problematic in cases of scientific misconduct.

## Specification of role-related responsibilities

One thing to learn from the discussion so far, we think, is that it is helpful if the contributions from different collaborators are stated beforehand as well as documented as the work proceeds. It is also valuable if it is made clear what roles different participants take on in collaborations. It would be a further improvement, we suggest, if it would be stated beforehand what responsibilities go with the different roles in research collaborations. Here policies at the university level, or perhaps at the national level, could support researchers in how to think and act responsibly in research collaboration, thus having a pedagogical role, as well as being a basis for holding participants responsible. Referring back to the terminology discussion, we could say that specified task-responsibilities relating to different roles in collaboration constitute the basis for moral responsibilities. These ‘check-lists’ of responsibilities would be preliminary in the sense that investigations into misconduct may reveal that there are acceptable exceptions in individual cases from the ordinary distribution of responsibility.

We suggest that specifications are needed at three levels:

1. greater clarity about the responsibilities tied to different roles in collaborations
2. greater clarity about what situations or events relating to actual or potential scientific misconduct call for action
3. greater clarity about what is the appropriate action(s) in relation to these different situations or events

There will be different kinds of responsibilities vis-à-vis research relating to one’s role in the project, but also relating to other arrangements, such as the supervision of a doctoral student. Being principal investigator or member of the steering group might bring a certain set of task responsibilities relating to the collaboration as a whole—and the corresponding moral responsibilities—that go well beyond what members of contributing groups have, while being supervisor to a doctoral student might bring specific responsibilities relating to this role, in addition to those relating to one’s role in the collaboration.

There may be many different kinds of situations and events concerning scientific misconduct or suspicion thereof that call for action in some respect. Here are some examples:

- The researcher obtains direct evidence of scientific misconduct, such as plagiarism or manipulation of data
- Results from collaborators look a bit too good to be true
- A disproportionate production by the collaborating partner, or the doctoral student/post doc, considering the concerned time period and other obligations
- Selective reporting of end-points, in particular if one or more of those originally identified as most important are no longer included
- Discussions or comments in passing regarding data collection, data washing, analyses, etc. where some procedures do not seem quite right
- Descriptions by others of procedures, data, analyses, etc., or use of images or graphs, that the researcher finds to be incorrect
- Exaggerations of results or their implications for future research or for practice

Many of the points listed here are no direct evidence of misconduct—an inclusion of an incorrect image in a PowerPoint presentation could be a simple mistake, the descriptions of procedures that were briefly overheard in the cafeteria could be unfair to what was actually done, and exaggerations of results may not be dramatic enough to be correctly described as misconduct. But leaving a series of hints like that unattended could be seen as problematic from the perspective of responsible collaboration, and co-authorship.

Responsibilities may shift depending on the development of events. If members of a research group suspect misconduct and therefore inform others, then the responsibility to do something also spreads. It may be the case that someone in a more senior function has a responsibility to monitor events in the lab, yet, if doctoral students come across aggravating information relating to misconduct, the most acute responsibilities lie with them.

The appropriate kind of action as a response to recognised or suspected misconduct by collaborating partners will partly depend on local policies. There are, however, some standard alternatives when it comes to reporting incidents: formally reporting the incidents, discussing them with your nearest superior and take it from there, reporting to the nearest superiors of the individuals you suspect, or reporting to the PI of the project, or to someone on the steering group. There are a number of other options besides reporting. For instance, those who are suspicious of others may want to confront them with the questions and accusations first. This would be the best option for e.g. the PI or the nearest superior. Sometimes it might be a good idea to call for a meeting to discuss the issues and try to sort things out, either with all collaborating partners or within a single research group.

While of considerable interest to further clarify the specific responsibilities tied to different roles in research collaborations merely sketched here, and provide any such claims with adequate argumentation, a satisfactory approach to responsibility for scientific publication would also have to involve both the scientific journals and the academic institutions. In particular, more work needs to be done regarding the responsibilities of individual universities. Part of this concerns the incentives to publish and how to publish. Another part concerns the attitude and culture fostered at our academic institutions. In order for progress to be made regarding much needed change of unhealthy research environments, concepts like ‘research culture’ need to be concretized, most likely by being divided into more easily handled aspects. Some interesting attempts have been made in this direction (Martinson et al. 2013).

## Conclusions

It is neither fair nor constructive to hold all co-authors on a fraudulent paper responsible for everything about it regardless of their involvement. Neither is it fair or particularly helpful to ascribe such responsibility to some specific individual. We have suggested that it can be much more constructive to specify what kinds of task responsibilities come with different roles in a project and describe what kinds of situations or events call for some kind of action, and what the appropriate actions might be. If part of a well-advertised local or national policy, task responsibilities can be turned into moral obligations.

## Footnotes

1 It should be noted that this is not the same position statement as the *Singapore Statement on Research Integrity*, which was also developed at the 2nd World Conference on Research Integrity. The one we discuss is more substantial and has more to say on accountability and responsibility.
